# Genes Regulated by Vitamin D in Bone Cells Are Positively Selected in East Asians

**DOI:** 10.1371/journal.pone.0146072

**Published:** 2015-12-31

**Authors:** Elena Arciero, Simone Andrea Biagini, Yuan Chen, Yali Xue, Donata Luiselli, Chris Tyler-Smith, Luca Pagani, Qasim Ayub

**Affiliations:** 1 The Wellcome Trust Sanger Institute, Wellcome Genome Campus, Hinxton, CB10 1SA, United Kingdom; 2 Department of Biological, Geological and Environmental Sciences, University of Bologna, 40126, Bologna, Italy; 3 Division of Biological Anthropology, University of Cambridge, CB2 1QH, Cambridge, United Kingdom; University of Iceland, ICELAND

## Abstract

Vitamin D and folate are activated and degraded by sunlight, respectively, and the physiological processes they control are likely to have been targets of selection as humans expanded from Africa into Eurasia. We investigated signals of positive selection in gene sets involved in the metabolism, regulation and action of these two vitamins in worldwide populations sequenced by Phase I of the 1000 Genomes Project. Comparing allele frequency-spectrum-based summary statistics between these gene sets and matched control genes, we observed a selection signal specific to East Asians for a gene set associated with vitamin D action in bones. The selection signal was mainly driven by three genes CXXC finger protein 1 (*CXXC1*), low density lipoprotein receptor-related protein 5 (*LRP5*) and runt-related transcription factor 2 (*RUNX2*). Examination of population differentiation and haplotypes allowed us to identify several candidate causal regulatory variants in each gene. Four of these candidate variants (one each in *CXXC1* and *RUNX2* and two in *LRP5*) had a >70% derived allele frequency in East Asians, but were present at lower (20–60%) frequency in Europeans as well, suggesting that the adaptation might have been part of a common response to climatic and dietary changes as humans expanded out of Africa, with implications for their role in vitamin D-dependent bone mineralization and osteoporosis insurgence. We also observed haplotype sharing between East Asians, Finns and an extinct archaic human (Denisovan) sample at the *CXXC1* locus, which is best explained by incomplete lineage sorting.

## Introduction

**Fig 1:**
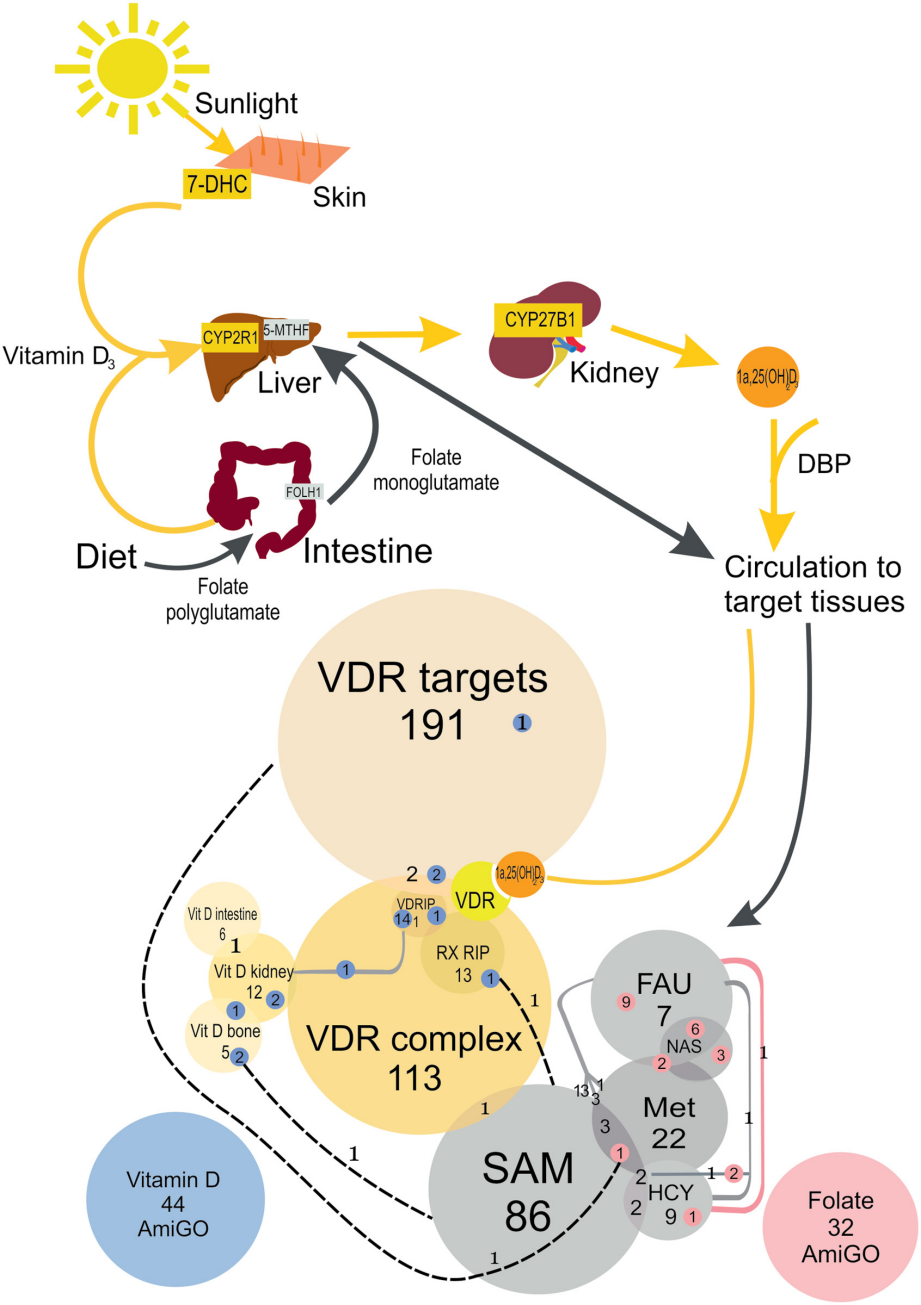
Vitamin D and folate acquisition, metabolism and gene sets analyzed in this study. The upper part shows metabolism of vitamin D (yellow arrows) and folate (black arrows). Vitamin D_3_ can be obtained from the diet, but it is mainly synthesised in the skin from 7-dehydrocholesterol (7-DHC) in response to light. It is then transported into the liver where it is hydroxylated to produce 25-hydroxyvitamin D_3_ which is subsequently converted into its active form 1α, 25- dihydroxy vitamin D_3_. This is transported in blood by vitamin D binding protein (DBP) and binds vitamin D receptor (VDR). The lower part shows gene sets analyzed in this study. The circles are proportional to number of genes in each set. The numbers in blue or pink circles indicate number of genes in each set that were present in AmiGO using the search terms “Vitamin D” (blue) or “Folate” (pink). Additional vitamin D (Vit D or VD) and folate (FA) gene sets are shown in shades of yellow and grey, respectively. The vitamin D gene sets that were generated included vitamin D targets identified by ChIP-Seq (VDR targets), genes involved in vitamin D action in bones, kidneys and intestines and all proteins involved in the VDR activation complex, including those directly interacting with VDR (VDRIP) and RXR (RXRIP) receptors. Folate gene sets include enzymes and receptors involved in dietary folate uptake and transport (FAU), proteins involved in nucleic acid synthesis (NAS) and methylation. The latter were sub-divided into genes involved in metabolism of methionine (Met), homocysteine (HCV) and S-adenosyl methionine methylation (SAM). The small blue and pink circles indicate the number of genes in the manually curated vitamin D and folate gene sets, respectively, that were also identified by AmiGO.

Fat-soluble vitamin D and water-soluble folate (folic acid, vitamin B9) are activated and degraded by ultraviolet (UV) radiation, respectively, and are necessary for human development and physiology [1–3]. These processes are controlled by many genes (Fig 1) that are thus likely to have been affected by the expansion of humans from tropical Africa into northern climes in the last 50–60,000 years, and may, therefore, have been targets of selection as humans adapted to new diets and environments. Seasonal variation in UV radiation at higher latitudes has been linked to natural selection in skin pigmentation of modern humans [3], and it has been suggested that lighter skin pigmentation was necessary to maintain homeostasis of vitamin D and protect against infections and skeletal deformities that are associated with reduced levels of this vitamin [3, 4]. Dietary intake of vitamin D is usually inadequate for normal physiological development and it is mainly produced in the skin by UV irradiation of 7- dehydrocholesterol. Its biologically active form (1α, 25-dihydroxyvitamin D3) acts as a hormone to regulate gene expression in several organs. It does so by binding the vitamin D receptor (VDR), which forms a heterodimer with retinoid X receptor (RXR), and recruits several other proteins to form the Vitamin D activation complex (Fig 1). Vitamin D3 regulates target gene expression in many tissues and has major roles in diverse physiological functions, being primarily responsible for calcium and phosphate homeostasis and bone remodeling [1]. In contrast, folate is exclusively obtained from the diet and is required for nucleic acid synthesis and repair, and methylation of DNA, proteins and fats [2, 5, 6].

Due to their crucial role in various metabolic pathways, a deficiency, or excessive intake of vitamin D or folate leads to a wide range of diseases. Vitamin D deficiency, which affects millions of people worldwide [7], has been associated with rickets, osteomalacia, autoimmune disorders and disorders of aging like osteoporosis, type 2 diabetes, cardiovascular diseases, and cancer [8, 9]. Folate deficiency during pregnancy has been associated with neural tube defects, anemia, cancer, cardiovascular and nervous system diseases. Folate also reduces homocysteine blood levels, thus acting indirectly as well to reduce risk of stroke, coronary and peripheral vascular diseases [5, 6]. Despite insufficient data on worldwide deficiency prevalence of these micronutrients, it is generally accepted that they have a substantial impact on human reproduction and mortality [10, 11].

In view of their functional importance and dependency on climate and diet, we set out to investigate signatures of adaptation in worldwide populations in gene sets associated with the function of these two vitamins. We used re-sequencing data from 13 worldwide populations generated by Phase I of the 1000 Genomes Project [12] (http://www.1000genomes.org) and applied an algorithm that we had developed earlier to test for evolutionary adaption in any chosen set of genes. This method compares three frequency-spectrum-based summary statistics (Tajima’s D [13], Fay and Wu’s H [14] and Nielsen’s *et*. *al*.’s composite likelihood ratio [15]) between user-generated gene sets and matched control genes using a sampled randomization test and can identify selection signals in a gene set when at least 10% of the genes in the list are under selection [16].

## Results

We ran the algorithm using the positive and negative controls from our previous study [16] to ensure that the selection signals could be identified in the 1000 Genomes Project Phase I data used here [12], as well as in the Pilot data used earlier [17]. As expected, we observed strong selection signals in non-African populations (S1 Fig) for the positive controls, and also in genes involved in the melanin pathway and candidate selected skin pigmentation genes obtained from the literature [18]. No selection signature was observed in any population for the negative controls or the list generated using the search term “pigmentation” in AmiGO Gene Ontology (GO) [19], because this long list included many genes not specifically related to skin pigmentation, illustrating the importance of an appropriate gene list in such tests. Similarly AmiGO gene lists were retrieved using the search terms “vitamin D” and “folate” and these did not yield any significant support for positive selection which led us to refine the lists according to the biochemical pathways and the putative targets of these two vitamins (Fig 1). The vitamin D gene sets that were generated included vitamin D targets identified by ChIP-Seq [20], genes involved in vitamin D action in bones, kidneys and intestines and all proteins involved in the formation of the VDR activation complex, including those directly interacting with VDR (VDRIP) and RXR (RXRIP) receptors (Fig 1 and S1 Table). Thus, the final lists for skin pigmentation, vitamin D and folate have been obtained through a careful analysis of their functions.

### Signatures of positive selection in vitamin D gene sets

**Fig 2:**
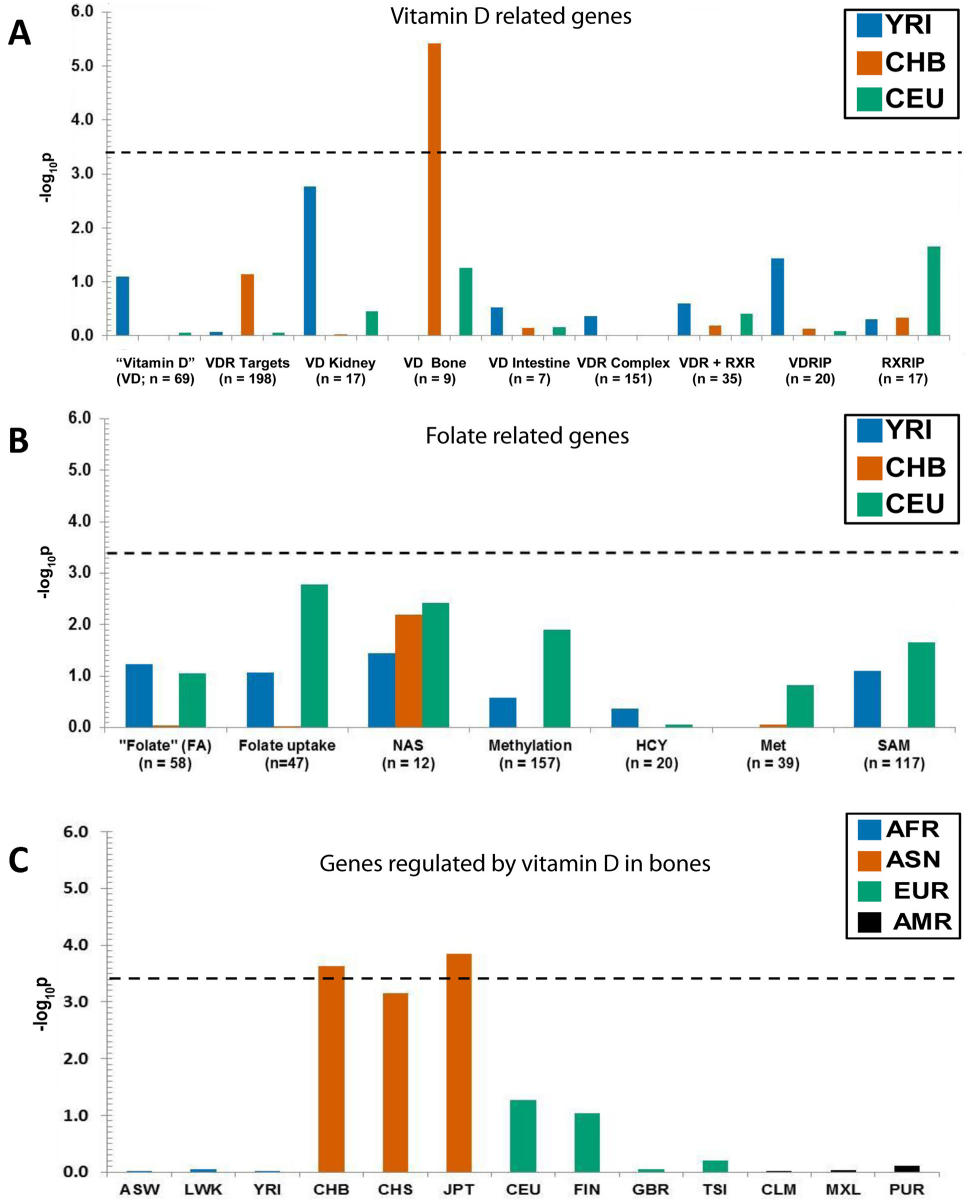
Positive selection in East Asians for genes regulated by vitamin D in bone. The y axis shows the −log_10_ of the combined p-value summarized from individual frequency-spectrum-based analysis on sets of Vitamin D (A) and Folate (B) related genes in three continental populations. The dashed horizontal line depicts the threshold of the −log_10_ p-value for multiple comparisons after applying the Bonferroni correction (13 populations x 9 gene sets). (C). Positive selection in East Asians for genes regulated by vitamin D in bone. African (AFR) populations included ASW (African Ancestry in Southwest USA), LWK (Luhya in Webuye, Kenya) and YRI (Yoruba in Ibadan, Nigeria). Asians (ASN) were represented by CHB (Han Chinese in Beijing, China), CHS (Han Chinese South China) and JPT (Japanese in Tokyo, Japan); Europeans (EUR) included CEU (Utah residents with ancestry from northern and western Europe), FIN (Finnish in Finland), GBR (British from England and Scotland, UK) and TSI (Tuscans in Italy). Americans (AMR) were CLM (Colombians from Medellin, Colombia), MXL (Mexican Ancestry in Los Angeles, California, USA) and PUR (Puerto Rican in Puerto Rico, USA).

Next, we examined several gene sets involved in vitamin D action (Fig 1 and S1 Table). Our strategy was to initially screen representative populations from the three continental regions (YRI—Yoruba in Ibadan, Nigeria representing Africans; CHB—Han Chinese in Beijing, China representing East Asians and CEU—Utah residents representing ancestry from northern and western Europe) using the AmiGO and refined gene sets, and subsequently all 13 Phase I 1000 Genomes Project populations for gene sets in which a selection signal was observed in YRI, CHB or CEU. As the selection pipeline used in this study was implemented using 1000 Genomes Project Pilot data comprising these three continental populations we initially sought to replicate the analyses on 1000 Genomes Project Phase 1 data using these three populations to show that the selection pipeline gave reliable and consistent results using these different 1000 Genomes datasets. Subsequently we extended our approach using all 13 worldwide populations from 1000 Genome Project data. Our aim was to identify signals that were common to continental populations and therefore, less likely to contain false positives. For all our analyses we used a stringent p-value threshold after applying the Bonferroni correction for multiple testing based on 13 populations, the only gene list showing a significant signature of selection was one that included genes involved in vitamin D action in bones (S3 Table and Fig 2A). This gene list showed significant enrichment in comparison with matched controls in the CHB, as well as the JPT, Japanese in Tokyo, Japan (Fig 2C), therefore highlighting a general pattern of selection over a broad geographic area. Compared to the CHB, the signal in the CHS (Han Chinese South China) reached borderline significance and was just below the Bonferroni-corrected threshold p-value. This can be explained due to Chinese population sub-structure [12, 21].

### No signatures of positive selection in folate gene sets

In contrast, folate gene sets (Fig 1 and S2 Table) yielded no signatures of positive selection overall, after adjusting for multiple comparisons, and we concluded that the genes associated with the folate pathway did not undergo selection detectable by our approach in any of the screened continental groups (S3 Table, Fig 2 and S2 Fig).

### Genes under positive selection

Overall, out of the 627 unique Ensembl protein coding genes in the vitamin D and folate gene sets (http://www.ensembl.org/index.html), we identified 19 outliers (S4 Table) in YRI, CHB or CEU with a combined p-value ≤ 0.03 (based upon Fay and Wu’s H [14] and Nielsen *et al*.’s composite likelihood ratio tests [15]) (S2 Fig). Tajima’s D test was not taken into account, since significant negative values of this statistic alone are also associated with purifying selection [16]. After correcting for multiple comparisons, the only gene list overcoming the threshold of significance is the one comprising genes regulated by vitamin D in bone tissue. Within this gene set, three genes, (CXXC finger protein 1, *CXXC1*; low density lipoprotein receptor-related protein 5. *LRP5* and runt-related transcription factor 2, *RUNX2*), were selected in all the East Asian populations. *RUNX2* also showed a signature of selection in the YRI. All three genes were examined in further detail to characterize disease associations and identify candidate variants associated with the putative selection signal detected by the tests based on allele frequency spectra.

### Disease association and phenotypes

To investigate the functional consequences of disruption of the selected genes, we mined the available phenotype data in Online Mendelian Inheritance in Man (OMIM—http://omim.org), genome-wide association study (GWAS) catalogue, and zebrafish and mouse model organisms [22–24]. Two of the three genes found to be under selection in East Asians are associated with abnormalities in human skeletal development. Variants in *LRP5* and *RUNX2* variants are also associated with bone mineral density and increased risk of osteoporosis, and osteoporotic fractures in association studies in Caucasians and Asian populations. A derived missense variant (*rs3736228*; p.Ala1330Val) in *LRP5* is of particular interest due to its association with bone mineral density and osteoporosis (OMIM: 166710) [25, 26]. The risk allele frequency of this associated variant is 20% in East Asians, and it lies within a 10 kb selected window in *LRP5*.

In mouse models *CXXC1* knock-outs exhibit embryonic lethality and developmental defects [27]. Mice homozygous for a targeted null mutation in *Lrp5* develop a low bone mass phenotype that becomes evident post-natally, and is secondary to decreased osteoblast proliferation and function in a *Cbfa1*-independent manner [28, 29]. These features recapitulate the osteoporosis-pseudoglioma syndrome (OMIM: 259770) in humans that is caused by *LRP5* inactivation. Mouse models of two human non-synonymous mutations in *LRP5* (ClinVar: *c*.*512G>T* (*p*.*Gly171Val*); and *c*.*641C>T* (*p*.*Ala214Val*)) replicate the high bone mass phenotype (OMIM: 601884) associated with these variants [30]. Mice homozygous for a *Runx2* null allele exhibit neonatal lethality and skeletal abnormalities particularly in clavicle and cranial bones, recapitulating the underdeveloped or absence of clavicles seen in cleidocranial dysplasia (OMIM: 119600) that has been associated with mutations in *RUNX2*.

### Signal refinement and functional annotations of selected genes

Using population differentiation and presence of extended haplotypes we pinpointed putative causal variants that could be responsible for the adaptation signal (S3 Fig). We examined variants in the genic region and 1 Mb up- and down-stream of each gene to look for regions with putative Vitamin D Response Element (VDRE). Variants that were present in these regions were classified as genic (those falling within the coding region of each gene) and non-genic (variants falling up or down-stream of a gene). Candidate functional genic variants were chosen if they overlapped any 10 kb positively-selected window, lay in DNAse I hypersensitivity regions and had an Ensembl functional annotation as determined by the Variant Effect Predictor tool [31]. Candidate non-genic variants were shortlisted if they overlapped a VDRE or an ENCODE annotation as determined by the Variant Effect Predictor tool. We identified three such regulatory variants in *CXXC1*, 11 in *LRP5* and 22 in *RUNX2* (S4 Fig). Seven *RUNX2* variants were identified in the YRI and 15 in the CHB. One variant each in *CXXC1* (*rs59393148*) and *RUNX2* (*rs2677100*) and two in *LRP5* (*rs671494* and *rs649772*) had high (≥ 72%) derived allele frequencies in East Asians, and fell in the 10 kb selected window and DNase hypersensitivity regions in cell lines, including osteoblasts, suggesting that these could be driving the selection signal (Table 1). We also identified two *RUNX2* regulatory variants with a higher derived allele frequency in Africans (*rs13201287* and *rs10948238*). An additional two regulatory intronic variants in *RUNX2* (*rs7751427* and *rs7771980*) were also associated with regulatory epigenetic features in osteoblasts and an active promoter regions in several cell lines, but were present at low (≤ 10%) derived allele frequency in all populations. However, none of the candidate variants were associated with any disease or metabolic trait in the GWAS catalogue [32].

**Table 1:** Candidate regulatory variants.

Gene	Pop^a^	VariantType	Position^b^	SNP ID	Variant Alleles^c^					DAF (%)^d^		
					Ref	Alt	Anc^c^	Den	Nea	AFR	ASN	EUR
*CXXC1*	CHB	5’UTR	18:47814249	*rs59393148*	T	C^e^	A	T	C	75	89	91
*LRP5*	CHB	Intronic	11:68209477	*rs671494*	A	C^e^	A	A	A	22	75	65
*LRP5*	CHB	Intronic	11:68209478	*rs649772*	C	A^e^	C	C	C	22	72	63
*RUNX2*	CHB/YRI	Intronic	6:45420847	*rs2677100*	C	T^e^	C	C	C	87	96	65
*RUNX2*	CHB/YRI	Intronic	6:45511541	*rs10948238*	C^e^	T	T	T	T	28	96	59
*RUNX2*	YRI	Intronic	6:45511945	*rs13201287*	G	A^e^	G	G	G	45	4	28

Gene name and the population in which the selection signal was observed are given along with the variant type, SNP ID, position, alleles and frequencies.

^a^ Populations YRI = Yoruba in Ibadan, Nigeria; CHB = Han Chinese in Beijing, China.

^b^ Variant position chromosome: genomic co-ordinates in human reference GRCh37.

^c^ Variant alleles Ref = reference; Alt = alternate; Anc = ancestral; Den = archaic Denisovan; Nea = Neanderthal. The ancestral and derived states of each variant were based on a 6 way primate alignment as determined by the Ensembl compara pipeline [50, 51].

^d^ Derived allele frequencies (DAF) AFR = Africans; ASN = East Asians; EUR = Europeans.

^e^ Derived alleles. For rs59393148 we consider the C allele to be derived in modern humans and Neanderthals, as the archaic Denisovan hominin is homozygous for the T allele which is also more prevalent in Africa.

### Haplotype Networks and putative archaic haplotype sharing

**Fig 3:**
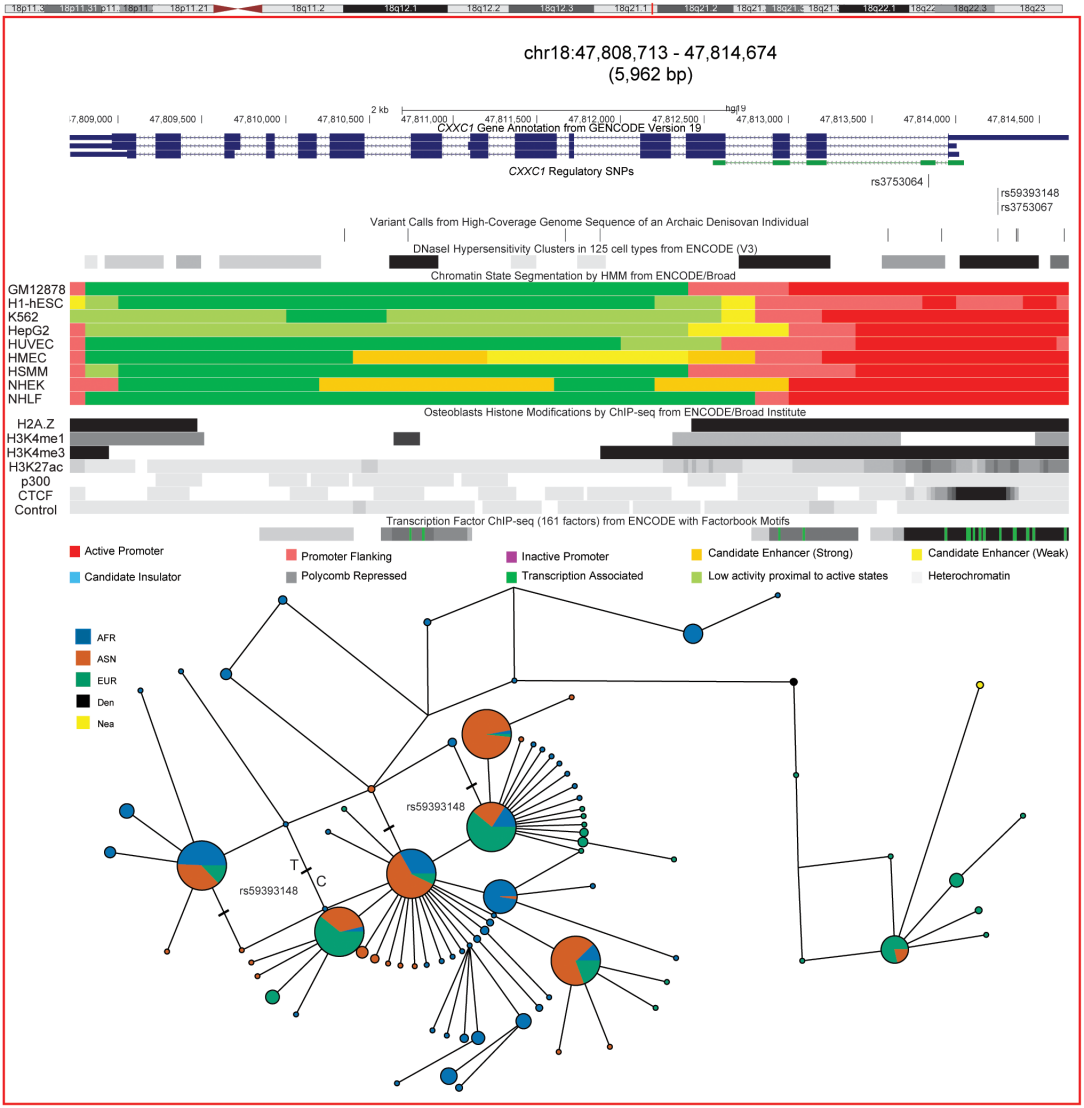
Positive selection at the *CXXC1* locus. A ~6 kb region on chromosome 18 that spans *CXXC1* showing GENCODE (Version 19) transcript annotation. The three short-listed candidate regulatory variants driving the selection signal in East Asians are all located in ENCODE annotated regions of open chromatin, depicted in the DNase I Hypersensitivity Clusters in 125 cell lines track, and show ENCODE chromatin state segmentation associated with an active promoter site in nine human cell lines. The latter include lymphoblastoids [GM12878]; embryonic stem cells [H1-hESC]; chronic myelogenous leukemia [K562]; hepatocellular carcinoma [HepG2]; umbilical vein endothelial [HUVEC]; mammary epithelial [HMEC]; skeletal muscle myoblast [HSMM]; skin epidermal keratinocytes [NHEK] and lung fibroblasts [NHLF]). Positions of histone modifications in osteoblasts are indicated by shaded bands and the black shade signifies enrichment. In osteoblasts the position of the histone sequence variant, H2A.Z, that determines accessibility of the transcription start site (TSS) and histone modifications like H3K4me3 that are enriched around TSS (dark bands) encompasses the candidate regulatory variant site and show binding for many transcription factors. H3K4me1 and H3K27ac modifications and p300 marks are enriched around active enhancers and CTCF indicates insulator regions. The lower part of the figure shows median joining haplotype networks in this region that is in high LD (r^2^ ≥ 0.95) in CHB. Phased haplotypes generated by the 1000 Genomes Project were used to construct this network. The derived C allele for the regulatory variant *rs59393148* lies on the branch leading towards the most frequent haplotype found in East Asians, and shows a star like expansion typical of a selection signal. Note the proximity of archaic human haplotypes with a subset of East Asian (ASN) and European Finnish samples. These samples lie on a divergent branch that is closer to the Neanderthal (Nea) and Denisovan (Den) haplotype when compared with the rest of the modern human population samples.

**Fig 4:**
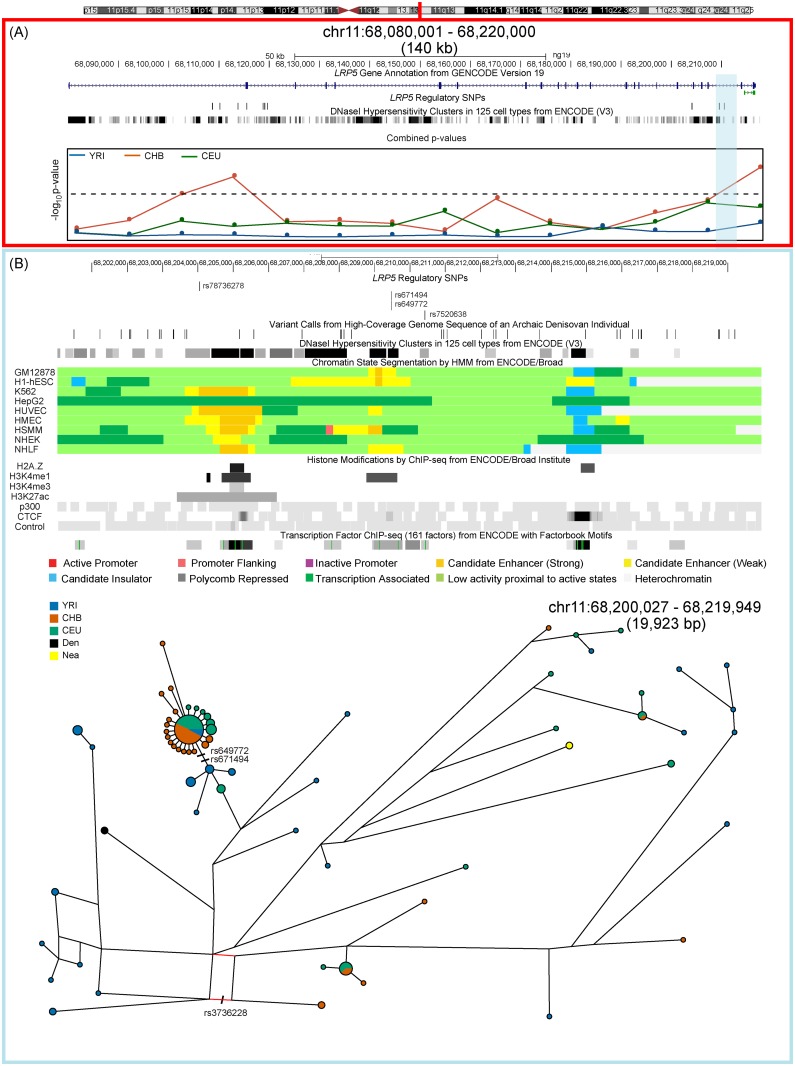
Positive selection at the *LRP5* locus. (A). A 140 kb region on chromosome 6 that spans *LRP5* showing GENCODE (Version 19) transcript annotation. Positions of 11 candidate regulatory variants and DNase I Hypersensitivity Clusters are shown along with the −log_10_ of the combined p-values from frequency-spectrum-based analysis in three continental populations. The significance threshold is indicated by the dashed line and two non-overlapping 10 kb windows have a significant combined p-value in CHB. (B). A closer look at the 3’ selected region in East Asians (highlighted in blue). The region contains both variants with the highest derived allele frequency in East Asians (*rs649772* and *rs671494*) that lie in a DNase I hypersensitivity cluster and show ENCODE chromatin state segmentation associated with enhancer binding in several cell lines. In osteoblasts the variants lie within the histone sequence variant, H2A.Z, that determines accessibility of the transcription start site (dark bands) and there are additional H3K4me1 and H3K27ac histone modifications upstream of the variant. The candidate regulatory variant site also shows binding for many transcription factors. The lower part of the panel shows median joining haplotype networks in a ~20 kb region that is in high LD (r^2^ ≥ 0.95) in CHB. Phased haplotypes generated by the 1000 Genomes Project were used to construct this network. The derived alleles for the regulatory variants *rs649772* and *rs671494* lie on the branch leading towards the most frequent haplotype found in East Asians and shows a star like expansion typical of a selection signal. The non-synonymous variant *rs3736228* (red line) that is associated with bone mineral density in genome wide association studies lies on a separate branch.

**Fig 5:**
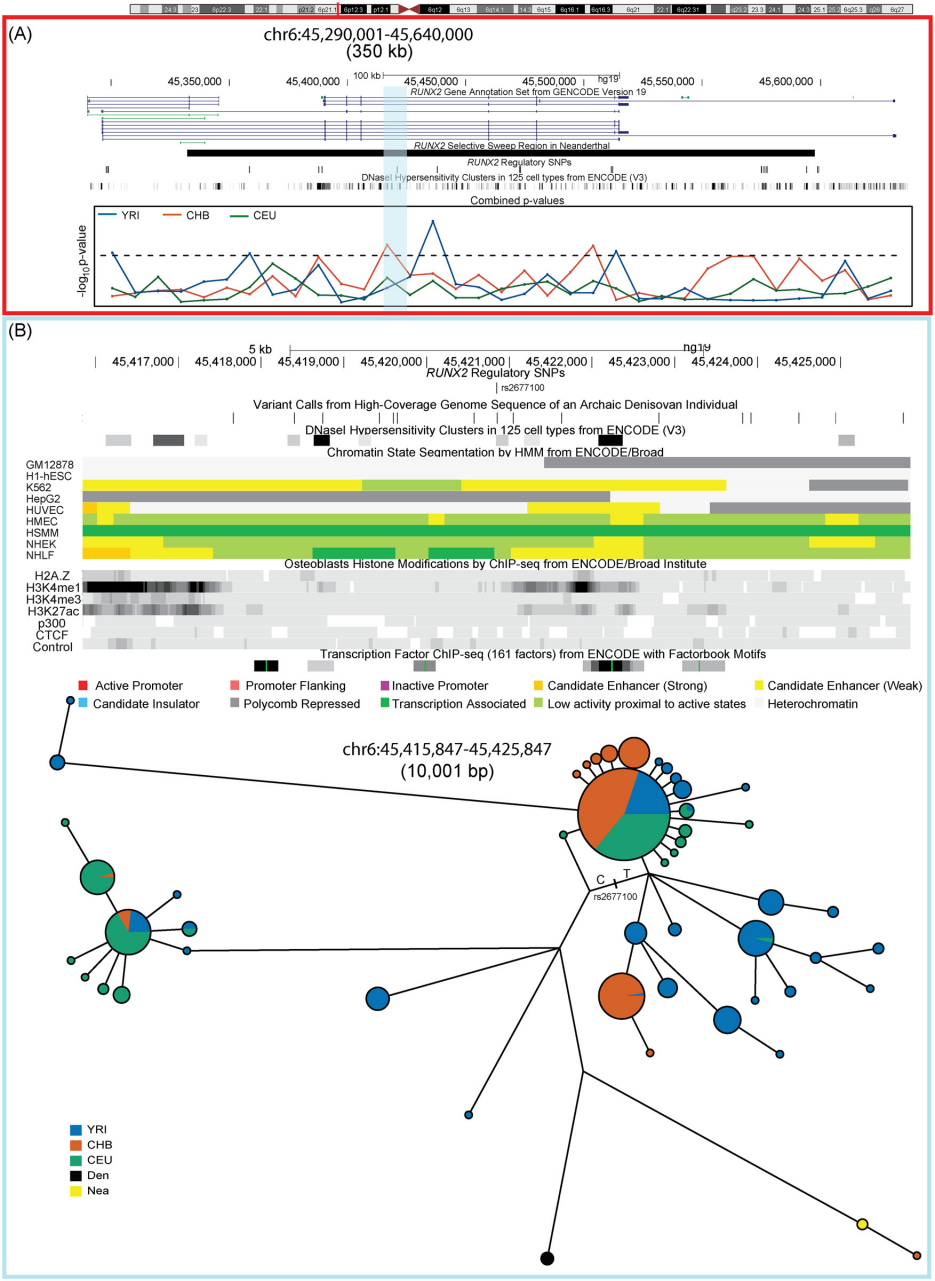
Positive selection at the *RUNX2* locus. (A). A 350 kb region on chromosome 6 that spans *RUNX2* showing GENCODE (Version 19) transcript annotation and the extent of the selective sweep region identified in the archaic Neanderthal genome. The positions of 22 candidate regulatory variants and DNase I Hypersensitivity Clusters are shown along with the −log_10_ of the combined p-values from frequency-spectrum-based analysis in three continental populations. The significance threshold is depicted by the dashed line and two non-overlapping 10 kb windows have a significant combined p-value in CHB and YRI. (B). A closer look at the first selected region in East Asians (highlighted in blue). The region contains the variant with the highest derived allele frequency in East Asians (*rs 2677100*) that overlies ENCODE chromatin state segmentation associated with an enhancer in K562 and is also associated with H3K4me1 and H3K27ac modifications in osteoblasts. The lower part of the panel shows median joining haplotype networks in a ~10 kb region that is in high LD (r^2^ ≥ 0.95) in CHB. Phased haplotypes generated by the 1000 Genomes Project were used to construct this network. The derived T allele for the regulatory variant *rs2677100* lies on the branch leading towards the most frequent haplotype found in East Asians and shows a star like expansion typical of a selection signal.

Median-joining haplotype networks for all three positively selected genes show high frequency haplotypes in East Asians. In all cases the derived allele for the putative candidate selected regulatory variant lay on the branch leading to the high frequency star-shaped haplotype cluster, characteristic of a selection signal (Figs 3–5). In the *CXXC1* haplotype network (Fig 3) the annotated regulatory variant that has the highest derived allele frequency in East Asians (*rs59393148*) lies on the branch leading towards the most frequent haplotype. This haplotype is shared with Europeans and Africans in whom it is present at a lower frequency. All three candidate regulatory variants lie in an active promoter region in numerous cell lines including osteoblasts (Fig 3). We also observed haplotype sharing between a subset of East Asian and Finnish samples and archaic (Neanderthal and Denisovan) hominins, which could represent either incomplete lineage sorting or archaic introgression. To rule out the latter, we estimated the length of this putatively introgressed haplotype and the number of differences between human and Denisova. The total length of haplotype sharing between these samples and Denisova was estimated to be ~25 kb, compatible with an introgression scenario as previously hypothesized [33]. However we detected a five-fold increase of single nucleotide differences between Denisova and the closest observed human haplotype (3 differences as opposed to the 0.6 expected under a simple mutation model after divergence). While these differences might be attributed to within-Denisova diversity, they also point toward incomplete lineage sorting as the simplest explanation for the haplotype sharing. Furthermore, each haplotype found within the median-joining networks for *CXXC1*, *LRP5* and *RUNX2* (Figs 3–5) was dated using the approach proposed by Voight *et*. *al*. [34] Assuming a generation time of 25 years the age of the most frequent East Asian star-like haplotypes in each gene are shown in Table 2. The selection events in East Asians occurred over a long time frame estimated at ~ 46, 26 and 13 thousand years ago for *RUNX2*, *CXXC1* and *LRP5* respectively, most likely after separation of the East Asian and European populations from a common non-African ancestor (Table 2).

**Table 2:** Age estimations (in years ± SD) of the selected haplotypes with star like genealogy in East Asians (ASN).

Gene	ASN
*CXXC1*	25,903 ± 2,213
*LRP5*	13,119 ± 3,521
*RUNX2*	46,530 ± 3,978

## Discussion

We have used re-sequencing data from 13 worldwide populations generated by the Phase I of the 1000 Genomes Project and an algorithm that we had developed earlier to test for evolutionary adaption in genes involved in the metabolism, regulation and action of vitamin D and folate. Both micronutrients are crucial to many metabolic processes as they play an important physiological role in all stages of development. Their dependency on sunlight and diet make them ideal candidates to study the genetic patterns of adaptation to the new environments that humans encountered on their way out of Africa. Furthermore, there is an overlap of four genes associated with metabolism, regulation and action of these vitamins which suggests that these interact as regulators of an integrated cellular machinery controlling gene expression [35]. We found evidence of positive selection in genes responsible for, or responsive to, action of vitamin D_3_ in bones in East Asians (Han Chinese and Japanese). No convincing selection signal was observed in any of the other gene sets including several analyzed folate gene lists.

Selection signals in the genes responsible for, or responsive to, action of vitamin D_3_ in bones in East Asians could be attributed to three genes, *CXXC1*, *LRP5* and *RUNX2*. All three lie in regions that have been characterized as being under positive selection in previous genomic selection scans, and in 1000 Genomes CHB populations [36] and contain at least one variant with a difference in derived allele frequency (ΔDAF) ≥ 0.5 among African and Eurasian continental populations [37–41]. The importance of these three genes can be also gauged from the fact that variants in two of the genes, *LRP5* and *RUNX2*, are associated with abnormalities in skeletal development in humans and mice [42, 43]. A missense variant (*rs121908669*; *c*.*511G>C* (*p*.*Gly171Arg*)) in *LRP5* is associated with autosomal dominant type 1 osteopetrosis (OMIM 607634), a disease associated with high bone density and distinct facial features like square jaw. A selection signal in *RUNX2* was also observed in Africans. *RUNX2* plays an important role in morphology of cranium and upper body skeleton and was also among the top 20 candidate selective sweep regions in the Neanderthal genome [44]. Nonsense and missense mutations in *RUNX2* have also been observed in several Chinese patients with cleidocranial dysplasia [45].

We used functional annotation, population differentiation and extent of linkage disequilibrium (LD) to identify several candidate regulatory variants in each gene that were most likely to be responsible for the adaptation signal we observed. All the candidate variants that we identified had the highest derived allele frequency in East Asians and lay in regions showing DNAse I hypersensitivity or ENCODE functional annotation in osteoblasts, supporting our conclusion that these could be responsible for the selection signal (Table 1). Simulations have shown that the window with the most significant frequency spectrum-based combined p-value lies within the 40 kb region surrounding the selected allele and that the Nielsen *et al*.’s CLR is better at localizing the selection signal due to selective sweeps and is robust to “assumptions regarding recombination rates and demography” [46]. All our candidate regulatory variants lay either within the selected 10 kb window (*CXXC1*) or were within 500–850 bp of the 10 kb window with the most significant combined p-value and were in high LD (r^2^ ≥ 0.8) with SNPs within the selected window (*LRP5* and *RUNX2*). These included one variant each in *CXXC1* (*rs59393148*) and *RUNX2* (*rs2677100*) and two in *LRP5* (*rs671494* and *rs649772*). The two *LRP5* variants were next to each other and in perfect LD suggestive of a multi-nucleotide polymorphism that might have arisen by a single mutation. A benign non-synonymous SNP (*rs3736228*) in *LRP5*, which is also in LD with our candidate variants, is another attractive candidate for the selection signal in East Asians. The derived *T* allele of this variant (*rs3736228*) has been associated with low bone mineral density and risk of osteoporosis. However, the derived *T* allele frequency in East Asians is ~ 20% and it seems unlikely to be the signal picked up by the frequency-spectrum-based tests. In addition, the two candidate regulatory variants in *LRP5* lie on the branch leading towards the selected haplotype in the median-joining haplotype network (Fig 4).

In comparison with the Africans and Europeans, the East Asians have very high derived allele frequencies for several candidate regulatory variants across these three loci. This enabled us to detect the signal of positive selection in populations from this (East Asia) region. However, as attested by the derived allele frequencies for the candidate loci and the haplotype networks, the most likely candidate variants responsible for these selection signals also have appreciable derived allele frequencies among the other two continental populations, particularly for *CXXC1* and *LRP5*. In Africans an independent signal for selection is also observed in *RUNX2* using the site frequency-spectrum-based tests and there are two intronic regulatory variants (*rs13201287* and *rs10948238*) in *RUNX2* with a higher derived allele frequency in Africa. These findings are in line with the expectation of new environments, diets and food availability acting on the genes associated with vitamin D action in bones and might shed light on alternate mechanisms of adapting to the decreasing availability of UV or dietary calcium.

The biochemical pathway for the production of the active form of vitamin D is fairly well conserved through evolution, thus highlighting the important role of this micronutrient in development and calcium homeostasis. This is also supported by our analyses as we did not detect any signal of selection in the genes involved in the synthesis of vitamin D. However, as vitamin D acts as a transcription factor regulating the expression of genes involved in different physiological processes, like bone mineralisation, different vitamin D targets may undergo selective pressures from various environmental stimuli. There is evidence that the active form of vitamin D and its receptor, VDR, evolved and specialized in the regulation of intestinal calcium absorption that is essential for proper mineralization of the skeleton particularly in environments that lacked sufficient intake of calcium. It is possible that these selective pressures lead to the evolution of target genes of vitamin D involved in osteoblast proliferation such as *CXXC1*, *LRP5* and *RUNX2*.

The evolution of light skin outside Africa has been associated with the synthesis of vitamin D in the skin, as the lack of this micronutrient can lead to the development of impairment that can affect human reproductive fitness [7, 47]. The lighter skin pigmentation could have evolved to facilitate homeostasis of vitamin D without affecting vitamin D specific genes. As modern humans moved out of Africa other independent selective pressures, such as new environments and dietary changes, may have affected specific vitamin D related genes (*CXXC1*, *LRP5* and *RUNX3*) with a direct action in bones. Taken together these results suggest that these could have evolved in parallel with the lighter skin colour for improving bone homeostasis and reproductive success. This is also suggested by the fact that none of the candidate of skin pigmentation selected loci are vitamin D targets. In addition, we observe no evidence for an overall selection signal in VDR targets identified by ChIP-Seq and none of the plausible candidate variants in the three selected loci (*CXXC1*, *LRP5* and *RUNX2*) lie in VDREs.

We also observed haplotype sharing between the Denisova, an archaic human specimen, and a subset of East Asian and European Finnish population samples (Fig 3) in a region surrounding a transcription factor binding site in the *CXXC1* genic region. Despite an increasing catalogue of archaic adaptive introgression in humans we conclude that incomplete lineage sorting seems the most parsimonious explanation for this observed haplotype sharing [48]. Functional characterization of selected candidate regulatory variants *in-vitro* or in animal models will be needed to interpret the phenotypic consequences of the selected alleles and provide a key to interpret the way selection pressures might have acted on these loci.

## Materials and Methods

### Data Sets

Protein coding gene sets for vitamin D (n = 44) and folate (n = 32) were initially generated by using the search terms “Vitamin D” and “Folate” in Homo sapiens in Ensembl (Ensembl Genes 72; GRCh37.p11) and AmiGO Gene Ontology [19], (http://amigo.geneontology.org/; 2013-07-27, version 1.8). These initial lists were subsequently refined by manual curation and subdivided into more specific sets according to the biochemical and functional properties of each gene (Fig 1). The vitamin D gene sets that were generated included vitamin D targets identified by ChIP-Seq [20]; genes involved in vitamin D action in bones, kidneys and intestines and all proteins involved in the VDR activation complex, including those directly interacting with VDR (VDRIP) and RXR (RXRIP) (Fig 1 and S1 Table). Four additional folate gene sets were generated to include the following categories: 1) Enzymes in the folate pathway; 2) Folate uptake proteins that included receptors involved in dietary folate uptake; 3) nucleic acid synthesis; and 4) methylation (S2 Table). The latter were sub-divided into genes involved in metabolism of methionine, homocysteine and S-adenosyl methionine methylation (SAM). Four genes were shared among the gene sets for vitamin D and folate. After removal of pseudogenes and non-autosomal genes a total of 417 genes were analyzed in the vitamin D and 214 in the folate gene sets (S1 and S2 Tables).

Positive and negative control gene sets described earlier [16] were used to confirm the reliability of the pipeline to detect signals of positive selection in low coverage whole genome sequences generated for 13 Phase I populations by the 1000 Genomes Project [12], since Pilot 1000 Genomes Project data had been used earlier [17]. The positive control gene set was collated from genes that lay in regions identified as being under positive selection in at least 7 genome-wide scans of selection, whereas the negative controls were generated from a list of protein coding genes that excluded those picked up in any genomic selection scan. Additional controls included sets of pigmentation genes that have been repeatedly picked up by numerous genomic selection scans as targets of positive selection. A pigmentation gene set (n = 116) identified by AmiGO was used and this set was further refined into separate lists of genes involved in skin pigmentation and melanin pathways. A published list of candidate positively selected skin pigmentation genes, that included several not identified by AmiGO search terms, was also used [18].

Genome-wide site frequency spectrum-based selection statistics were used to assess the selection signal. Tajima’s D [13], Fay and Wu’s H [14] and Nielsen *et al*.’s composite likelihood ratio values [15] were generated for non-overlapping ~10 kb windows from low-coverage sequence data obtained from 13 populations (1,080 individuals) from the 1000 Genomes Project. The Iberian populations in Spain (IBS) were excluded because of their low sample size.

### Analysis Pipeline

Briefly, for each of the gene sets a matched list of control genes was generated from the Ensembl database and frequency-spectrum-based summary statistics compared using a sampled randomization test as described earlier [16]. The analysis pipeline was implemented using Perl and R scripts and the input data consisted of stable Ensembl gene ID numbers organized into the various gene sets described above. For each gene in the input list the analyses pipeline generated 1,000 matched control protein coding genes from the Ensembl database. Three parameters, namely the gene GC content, length and recombination rate were used as the matching criteria and the 1,000 genes with the least Euclidean distance based on these three parameters were taken as matched controls. One of these matched genes was then randomly chosen as a match for the control list and for each input gene set 1,000 control sets were also generated. For detecting signals of positive selection the frequency-spectrum-based test statistic values were compared between each input gene set and its matched control for each population across the non-overlapping 10 kb windows. The p-values from the individual tests were summarized as a combined p-value using Fisher’s method for combining probabilities as described earlier [16]. In order to reduce the false positive rate, and in a modification from the earlier study, we estimated combined p-values using only the Fay and Wu’s H and Nielsen *et al*.’s composite likelihood ratio, because negative values of Tajima’s D are also indicative of purifying selection.

The pipeline was initially run to detect signals of positive selection in three populations, YRI, CHB and CEU, derived from the African, East Asian and European continental populations, respectively. Gene sets that showed evidence of selection in either of YRI, CHB or CEU groups were subsequently analyzed in all 13 populations. Selection signals for individual genes were identified based upon whether they contained any 10 kb windows with a combined p-value ≤ 0.03 obtained from the Fay and Wu’s H and Nielsen *et al*.’s composite likelihood ratio tests.

### Functional Annotations of selected genes

Genes with significant combined p-values (≤ 0.03, chosen to be more conservative than the standard 0.05) were also analyzed with a custom Perl script for several additional parameters to identify single nucleotide variants (SNVs) that were highly differentiated among the 1000 Genomes populations, as well as to identify putative functional variants. The aim of this downstream analysis was to flag selected genes with variants of interest. The criteria on which we chose these were based on genetic diversity (derived allele frequency) [41], population differentiation (F_ST_ and ΔDAF) and the extent of linkage (LD length) and number (LD number) of SNPs included in LD blocks defined by r^2^ ≥ 0.8, calculated with PLINK [49], surrounding each examined SNP. The ancestral and derived states of each variant were determined by the Ensembl compara pipeline [50, 51]

We also focused on 1 Mb up and down stream of each gene, using two sets of thresholds for the genic (variants within the coding region) and non-genic variants. The genic variants were shortlisted when showing the following parameters: Derived allele frequency ≥ 0.05; LD length and number ≥ 0; F_ST (CEU-CHB)_ ≥ 0.016; F_ST (YRI-CHB)_ ≥ 0.032; ΔDAF (CEU-CHB) ≥ 0.042; ΔDAF (YRI-CHB) ≥ 0.070. SNPs falling either within a positively selected ~10 kb window and ENCODE DNAse I hyper-sensitivity cluster were functionally annotated (S3 Fig). The shortlisted non-genic variants identified in this manner were further refined by application of a more stringent filter based on derived allele frequency (≥ 0.2) and with the additional condition of falling within a vitamin D response element (VDRE). VDREs were identified on the DNA sequence retrieved from UCSC browser for each gene (± 1 Mb) using a custom Perl script. Consensus VDRE binding sites were obtained from a previous study [52].

The SNPs that, for a given population, passed all the thresholds were functionally annotated using the Ensembl Variant Effect Predictor tool [31] and HaploReg (http://www.broadinstitute.org/mammals/haploreg/haploreg.php), a tool for annotation of regulatory variants in haplotype blocks. Genes and variants were also characterized by their presence in human disease databases such as Online Mendelian Inheritance in Man (OMIM http://omim.org), overlap with any variant trait associations with p-value 5.0 X 10–8 in the Genome Wide Association Studies catalogue (http://www.genome.gov/gwastudies) and Clinical Genomic Database (CGD), to understand the potential biological basis for selection. CGD is a manually curated database that includes ~2,700 genes causing human diseases culled from OMIM and the Human Gene Mutation Database Professional. In addition, phenotypes for knock-out zebrafish were obtained from the Zebrafish Mutation Project (http://www.sanger.ac.uk/Projects/D_rerio/zmp/) and those for the mouse from the International Knockout Mouse Consortium and International Mouse Phenotyping Consortium (http://www.knockoutmouse.org/) [22],[23].

### Haplotype Networks

Phylogenetic haplotype network analysis of genic regions showing multiple signatures of selection in one or more populations were carried out using the phased data from the 1,000 Genomes Project and the high coverage archaic genomes [53, 54]. For each selected gene we established the region in high LD (r^2^ ≥ 0.8 or D’ = 1) in East Asians (the population with the lowest combined p-value) and retrieved haplotypes using custom Python and Perl scripts for the African and Eurasian populations from the 1000 Genomes Project Phase I vcf files and two high coverage archaic hominids, Denisovan and Neanderthals [53, 54]. These haplotypes were subsequently used to generate median-joining networks for *CXXC1*, *LRP5* and *RUNX2* [55]. Age estimations of the selected haplotypes with star like genealogy was carried out as described earlier assuming a generation time of 25 years. The method estimates age from the probability that two chromosomes are homozygous at a given recombination distance from the selected site.

## Supporting Information

S1 Fig Skin pigmentation gene sets. (A) Pigmentation gene sets include those identified by AmiGo using search terms “Pigmentation”, “Melanin” or “Skin pigmentation”. Skin pigmentation candidates gene set was obtained from the literature. (B) Positive selection in pigmentation gene sets. Positive (P) and negative (N) control sets were those used previously [16]. The positive controls were collated from genes that lay in regions identified as being under positive selection in at least 7 genome-wide scans of selection, whereas the negative controls were generated from a list of protein coding genes that excluded those picked up in any published genomic selection scan. The y axis shows the −log_10_ of the combined p-value summarized from individual frequency-spectrum-based analysis. The dashed horizontal line depicts the threshold for multiple comparisons after applying the Bonferroni correction (3 populations x 7 gene sets).
(TIF)

S2 Fig No selection in a folate gene set in 13 populations. No selection signal was seen in the gene set comprising folate uptake proteins, including receptors involved in dietary folate uptake. The y axis shows the −log10 of the combined p-value summarized from individual frequency-spectrum-based analysis. The dashed horizontal line depicts the threshold for multiple comparisons after applying the Bonferroni correction (3 populations x 9 gene sets). African (AFR) populations included ASW (African Ancestry in Southwest USA), LWK (Luhya in Webuye, Kenya) and YRI (Yoruba in Ibadan, Nigeria). Asians (ASN) were represented by CHB (Han Chinese in Beijing, China), CHS (Han Chinese South China) and JPT (Japanese in Tokyo, Japan); Europeans (EUR) included CEU (Utah residents with ancestry from northern and western Europe), FIN (Finnish in Finland), GBR (British from England and Scotland, UK) and TSI (Tuscans in Italy). Americans (AMR) were CLM (Colombians from Medellin, Colombia), MXL (Mexican Ancestry in Los Angeles, California, USA) and PUR (Puerto Rican in Puerto Rico, USA).
(TIFF)

S3 Fig List of selected genes. Twenty of the 627 genes in the vitamin D and folate gene sets were outliers in YRI, CHB or CEU based on the frequency-spectrum-based tests. Four significant outliers were observed in the vitamin D and 15 in the folate set. One gene was common between the two sets. Ensembl gene id, HGNC gene symbol, gene size and knock-out mouse phenotypes are given. Colored cells in the vitamin D and folate columns indicate presence of significant 10 kb windows with a combined p-vaule ≤ 0.03. The color indicates the continental population in which these significant windows were observed. Africa = blue; East Asia = orange; Europe = green.
(TIF)

S4 Fig Schema showing analysis pipeline for signal refinement and identifying candidate functional variants. For each gene we examined the whole gene length (A) and 1 Mb up and down stream of each gene. Genes (A) with highly significant 10 kb windows (combined p-values ≤ 0.03) were analyzed for presence of highly differentiated single nucleotide polymorphisms (SNPs) with derived allele frequency ≥ 0.05 (F_ST (CEU-CHB)_ ≥ 0.016; F_ST (YRI-CHB)_ ≥ 0.032; ΔDAF CEU-CHB ≥ 0.042; ΔDAF YRI-CHB ≥ 0.070). From this we short-listed variants that lay within any significant 10 kb windows in *CXXC1*, *LRP5* and *RUNX2* (B). The number of variants increased for *CXXC1* because the selected window was larger than the gene size (5,962 bp). Finally we curated variants that were in regions of DNase I hypersensitivity (C) or vitamin D response elements (D). The extent of linkage (LD length) and number of SNPs (LD number) included in LD blocks was defined by r^2^ ≥ 0.8. Short listed variants identified in this manner were further refined by application of a more stringent filter based on derived allele frequency ≥ 0.2. The thresholds used for each analyses are shown and the table shows the number of short-listed candidates after application of each filter.
(TIF)

S5 Fig Candidate regulatory variants and DNase I hypersensitivity clusters in *CXXC1*, *LRP5* and *RUNX2*. Green and orange filled cells indicate the tissues and DNase I hypersensitivity cluster regions, respectively, that are associated with an ENCODE functional annotation for each SNP that was short listed. Candidate regulatory variant rs ids and positions (in build GRCh37) are shown.
(TIF)

S1 Table Vitamin D gene sets. (XLSX)

S2 Table Folate gene sets. (XLS)

S3 Table Summary statistics of frequency-spectrum-based neutrality tests in vitamin D, folate and control gene sets. (XLS)

S4 Table Genes showing positive selection signals. (XLSX)
