# Supplementary figures and images for: Genes Regulated by Vitamin D in Bone Cells Are Positively Selected in East Asians
Source: PLoS One. 2015 Dec 31;10(12):e0146072. doi: 10.1371/journal.pone.0146072 (PMC4697808; doi:10.1371/journal.pone.0146072)

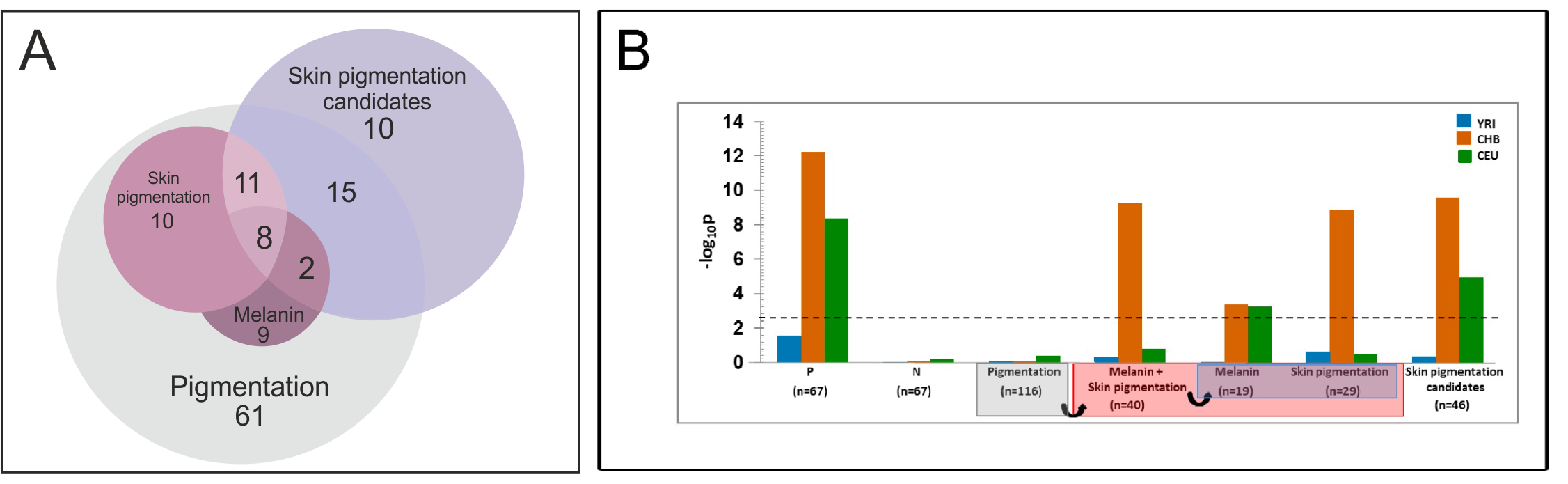

Supplement: S1 Fig — (A) Pigmentation gene sets include those identified by AmiGo using search terms “Pigmentation”, “Melanin” or “Skin pigmentation”. Skin pigmentation candidates gene set was obtained from the literature. (B) Positive selection in pigmentation gene sets. Positive (P) and negative (N) control sets were those used previously [16]. The positive controls were collated from genes that lay in regions identified as being under positive selection in at least 7 genome-wide scans of selection, whereas the negative controls were generated from a list of protein coding genes that excluded those picked up in any published genomic selection scan. The y axis shows the −log10 of the combined p-value summarized from individual frequency-spectrum-based analysis. The dashed horizontal line depicts the threshold for multiple comparisons after applying the Bonferroni correction (3 populations x 7 gene sets). (TIF) [file pone.0146072.s001.tif]

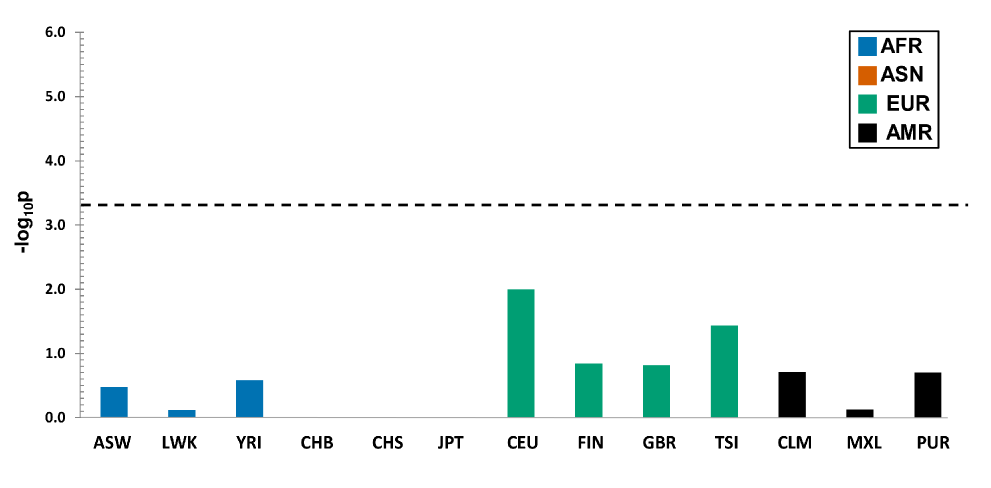

Supplement: S2 Fig — No selection signal was seen in the gene set comprising folate uptake proteins, including receptors involved in dietary folate uptake. The y axis shows the −log10 of the combined p-value summarized from individual frequency-spectrum-based analysis. The dashed horizontal line depicts the threshold for multiple comparisons after applying the Bonferroni correction (3 populations x 9 gene sets). African (AFR) populations included ASW (African Ancestry in Southwest USA), LWK (Luhya in Webuye, Kenya) and YRI (Yoruba in Ibadan, Nigeria). Asians (ASN) were represented by CHB (Han Chinese in Beijing, China), CHS (Han Chinese South China) and JPT (Japanese in Tokyo, Japan); Europeans (EUR) included CEU (Utah residents with ancestry from northern and western Europe), FIN (Finnish in Finland), GBR (British from England and Scotland, UK) and TSI (Tuscans in Italy). Americans (AMR) were CLM (Colombians from Medellin, Colombia), MXL (Mexican Ancestry in Los Angeles, California, USA) and PUR (Puerto Rican in Puerto Rico, USA). (TIFF) [file pone.0146072.s002.tiff]

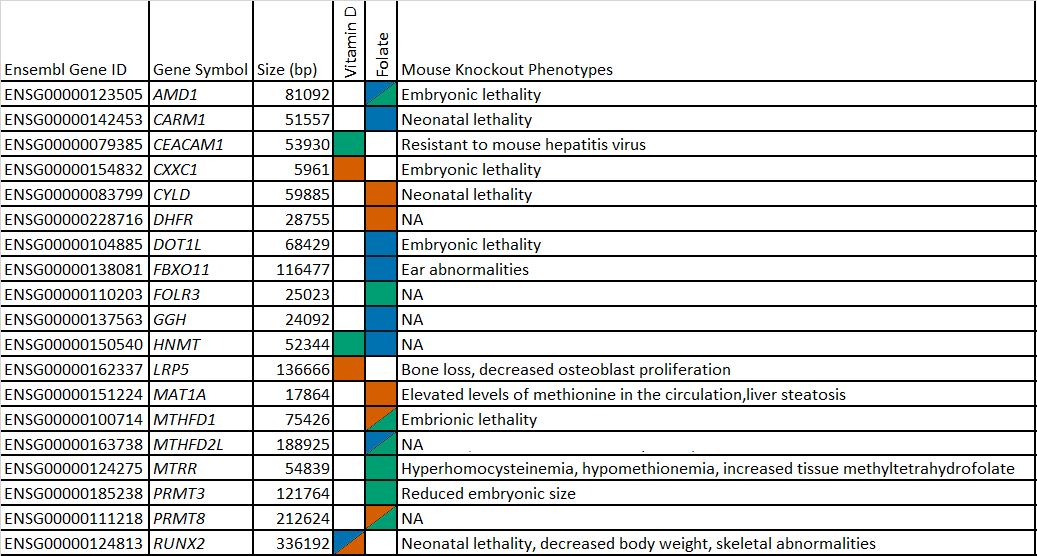

Supplement: S3 Fig — Twenty of the 627 genes in the vitamin D and folate gene sets were outliers in YRI, CHB or CEU based on the frequency-spectrum-based tests. Four significant outliers were observed in the vitamin D and 15 in the folate set. One gene was common between the two sets. Ensembl gene id, HGNC gene symbol, gene size and knock-out mouse phenotypes are given. Colored cells in the vitamin D and folate columns indicate presence of significant 10 kb windows with a combined p-vaule ≤ 0.03. The color indicates the continental population in which these significant windows were observed. Africa = blue; East Asia = orange; Europe = green. (TIF) [file pone.0146072.s003.tif]

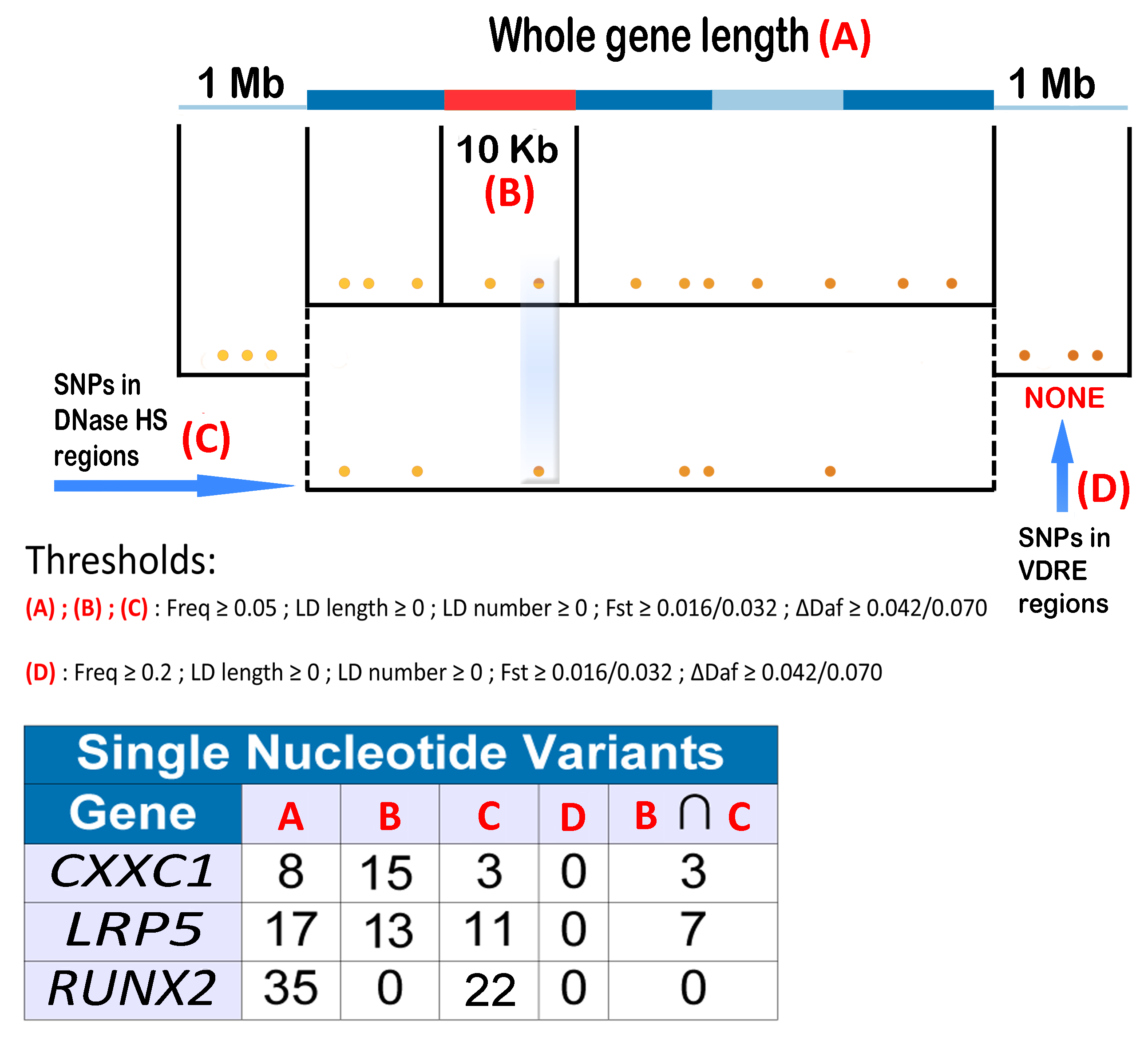

Supplement: S4 Fig — For each gene we examined the whole gene length (A) and 1 Mb up and down stream of each gene. Genes (A) with highly significant 10 kb windows (combined p-values ≤ 0.03) were analyzed for presence of highly differentiated single nucleotide polymorphisms (SNPs) with derived allele frequency ≥ 0.05 (FST (CEU-CHB) ≥ 0.016; FST (YRI-CHB) ≥ 0.032; ΔDAF CEU-CHB ≥ 0.042; ΔDAF YRI-CHB ≥ 0.070). From this we short-listed variants that lay within any significant 10 kb windows in CXXC1, LRP5 and RUNX2 (B). The number of variants increased for CXXC1 because the selected window was larger than the gene size (5,962 bp). Finally we curated variants that were in regions of DNase I hypersensitivity (C) or vitamin D response elements (D). The extent of linkage (LD length) and number of SNPs (LD number) included in LD blocks was defined by r2 ≥ 0.8. Short listed variants identified in this manner were further refined by application of a more stringent filter based on derived allele frequency ≥ 0.2. The thresholds used for each analyses are shown and the table shows the number of short-listed candidates after application of each filter. (TIF) [file pone.0146072.s004.tif]

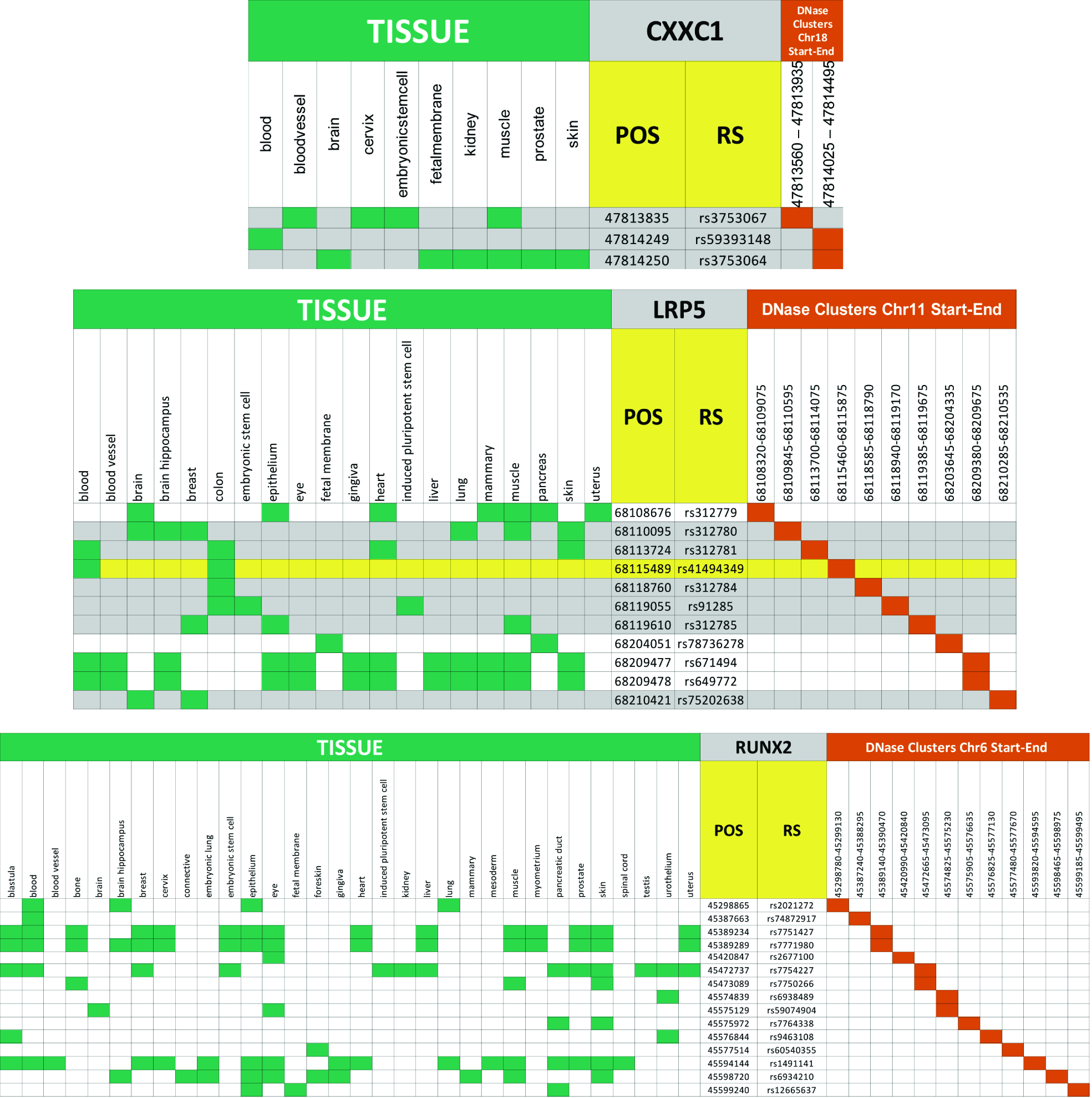

Supplement: S5 Fig — Green and orange filled cells indicate the tissues and DNase I hypersensitivity cluster regions, respectively, that are associated with an ENCODE functional annotation for each SNP that was short listed. Candidate regulatory variant rs ids and positions (in build GRCh37) are shown. (TIF) [file pone.0146072.s005.tif]
